# Cyanobacterial Metabolite Calothrixins: Recent Advances in Synthesis and Biological Evaluation

**DOI:** 10.3390/md14010017

**Published:** 2016-01-12

**Authors:** Su Xu, Bhavitavya Nijampatnam, Shilpa Dutta, Sadanandan E. Velu

**Affiliations:** Department of Chemistry, University of Alabama at Birmingham, 901, 14th Street South, Birmingham, AL 35294-1240, USA; suxu@uab.edu (S.X.); snij@uab.edu (B.N.); shilpad@uab.edu (S.D.)

**Keywords:** marine natural product, calothrixin, cyanobacteria, *calothrix*, antimicrobial activity, anticancer activity, total synthesis

## Abstract

The marine environment is host to unparalleled biological and chemical diversity, making it an attractive resource for the discovery of new therapeutics for a plethora of diseases. Compounds that are extracted from cyanobacteria are of special interest due to their unique structural scaffolds and capacity to produce potent pharmaceutical and biotechnological traits. Calothrixins A and B are two cyanobacterial metabolites with a structural assembly of quinoline, quinone, and indole pharmacophores. This review surveys recent advances in the synthesis and evaluation of the biological activities of calothrixins. Due to the low isolation yields from the marine source and the promise this scaffold holds for anticancer and antimicrobial drugs, organic and medicinal chemists around the world have embarked on developing efficient synthetic routes to produce calothrixins. Since the first review appeared in 2009, 11 novel syntheses of calothrixins have been published in the efforts to develop methods that contain fewer steps and higher-yielding reactions. Calothrixins have shown their potential as topoisomerase I poisons for their cytotoxicity in cancer. They have also been observed to target various aspects of RNA synthesis in bacteria. Further investigation into the exact mechanism for their bioactivity is still required for many of its analogs.

## 1. Introduction

### 1.1. Marine Natural Products

Dating back to ancient civilizations, natural products have played a vital role in drug discovery. Modern day drugs such as penicillin, morphine, and paclitaxel (Taxol™), which are used for the treatment of bacterial infections, pain, and cancer, respectively, are examples of natural products that have successfully progressed through the drug development pipeline. Natural products are abundant in several sources and are routinely extracted from plants, microorganisms, and animals. While terrestrial plants and terrestrial microbes continue to be the primary contributors to the natural product derived drug market, marine sources are an emerging resource that has been relatively unexplored until recent times [1]. Marine organisms are considered to yield superior natural products compared to terrestrial organisms in terms of novelty of structures and in producing potent bioactivities, due to the distinctive environmental conditions in which marine organisms live [2,3,4]. Factors such as predation, competition for space on highly populated coral reefs, and biochemical warfare between organisms have greatly contributed to the evolution of compounds with unique structures and potent biological effects [5].

### 1.2. Cyanobacteria

Microbial organisms in the marine environment have increasingly become one of the major focal points for investigations seeking to identify new chemical entities with novel structural backbones and diverse biological activities. In the 1970s, Professor Richard E. Moore at the University of Hawaii began exploring the chemistry of marine cyanobacteria [6]. Cyanobacteria are one of the oldest organisms on Earth, with an existence record of at least 2.7 billion years [7]. These prokaryote organisms are also known as blue green algae, cyanoprokaryotes, and cyanophytes due to their blue-green pigment, c-PC (c-phycocyanin) [8]. This pigment is used for photosynthesis and is considered to have played a crucial role in releasing oxygen into the primitive atmosphere [7,9]. Cyanobacteria possess a broad geographical distribution, ranging from limnic and marine environments to terrestrial habitats [10,11].

**Figure 1:**
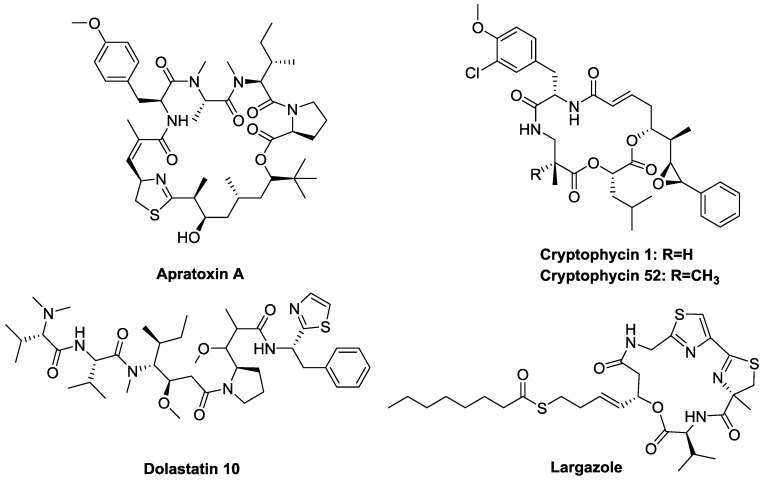
Biologically active natural products isolated from marine cyanobacteria.

A subclass of marine natural compounds is produced by cyanobacteria. The most studied species of marine cyanobacteria include *Nostoc*, *Calothrix*, *Lyngbya*, and *Symploca* [12]. Cyanobacteria are known to produce potent toxins. Several studies have investigated the pharmaceutical and biotechnological potential of the secondary metabolites isolated from cyanobacteria. These studies revealed a wide range of potent pharmacological effects that include anti-inflammatory, antimalarial, antiprotozoal, antimicrobial, immunosuppressant, anticancer, anti-HIV, antibacterial, anticoagulant, antifungal, anti-tuberculosis, antiviral, and antitumor activities [12,13,14]. A number of compounds that were isolated from aquatic cyanobacteria have showed promise as anticancer leads. Examples of such promising compounds include apratoxin A, cryptophycin, dolastatin 10, and largazole (Figure 1).

Apratoxin A is a cyclodepsipeptide derivative of the apratoxin family of cytotoxins isolated from a *Lyngbya* sp. collected from Guam [15,16]. It induces G_1_ phase cell cycle arrest and apoptosis, and has exhibited IC_50_ values ranging from 0.36 to 0.52 nM *in vitro* against 60 human tumor cell lines. However, *in vivo* studies revealed only marginal activity against early stage adenocarcinoma [17,18,19]. Another class of highly potent anticancer agents produced by cyanobacteria is cryptophycins. For example, cryptophycin 1, which was isolated from *Nostoc* sp. GSV224, has an IC_50_ of 5 pg/mL against KB human nasopharyngeal cancer cells, and 3 pg/mL against LoVo human colorectal cancer cells [20]. Its mechanism of action is through the suppression of microtubule dynamics, thereby inhibiting cells in G_2_/M phase. Cryptophycin 52, a chemical analog of cryptophycin 1, entered clinical trials but produced only marginal activity [21].

Dolastatin 10 is cyanobacterial metabolite that was synthesized by Pettit *et al.* [22,23] in the 1980s and then its origin was later confirmed when its direct isolation occurred from *Symploca* sp. Dolastatin 10 binds to tubulin on the rhizoxin-binding site and is an established antiproliferative agent that affects microtubule assembly, which leads to cell death during the G_2_/M phase [24]. In 2005, the efficacy and toxicity of dolastatin 10 were investigated in Phase II clinical trials in patients with advanced prostate cancer but it was discontinued due to the development of peripheral neuropathy in 40% of the patients [25]. Many research groups have sought after SAR studies of the synthetic analogs of this compound, which are now classified as auristatins. This group of compounds showed the most amount of promise as antibody drug conjugates. For example, brentuximab vedotin (adcetris, Seattle Genetics ) is now a FDA-approved drug against lymphoma. This compound is currently undergoing phase 3 studies to study its effect on cutaneous T-cell lymphoma, B-cell lymphomas, and mature T-cell lymphomas [26,27].

The cyclic depsipeptide largazole, of the genus *Symploca*, is a marine natural product that contains a methylthiazoline linked to a thiazole, as well as a 3-hydroxy-7-mercaptohept-4-enoic acid unit, and a thioester, which had previously not been observed in marine cyanobacterial natural products. This compound has been shown to selectively target transformed over non-transformed cells [28,29], through the inhibition of class I histone deacetylases (HDACs) [30,31]. Largazole’s interesting structure and biological activity have attracted strong interest from the synthetic chemistry community, which seeks to establish synthetic routes to largazole and to investigate its potential as a cancer therapeutic [32,33].

Thus, cyanobacterial metabolites have the potential for expanded utilization in drug discovery. Despite their potent biological activities, very few cyanobacterial compounds have entered clinical trials; one of the reasons is the complexity of synthesis of the natural products. The focus of this review is the natural products called calothrixins, with an emphasis on their synthesis and bioactivities.

### 1.3. Calothrixins

**Figure 2:**
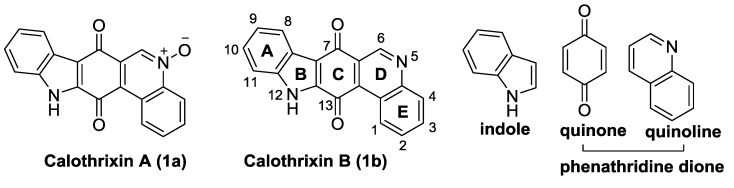
Structures of calothrixins.

Calothrixins A and B (Figure 2) are two cyanobacterial metabolites that were first isolated from *Calothrix* in 1999 by Rickards *et al.* [34]. Briefly, lyophilized cells of *Calothrix* strains were extracted with dimethyl sulfoxide (DMSO) and then with ethyl acetate (AcOEt) using Soxhlet extraction conditions. These extracts were fractionalized using a combination of bioassays, differential solubility, and chromatography, which yielded the relatively insoluble calothrixin A (1a) and its more soluble co-metabolite, calothrixin B (1b). Both structures were elucidated by Electron Impact Mass Spectra (EIMS), ^1^H, ^13^C and ^1^H–^1^H Correlation Spectroscopy (COSY) NMRs. Calothrixins possess an unique indolo[3,2-*j*]phenanthridine framework with an assembly of quinoline, quinone, and indole pharmacophores. Calothrixin B is generally known to be the neutral analog while calothrixin A is known to be its *N*-oxide analog.

These scaffolds have attracted the interests of organic chemists and biologists alike due to their unique structures and the promise they hold as lead compounds for cancer drug discovery. Herein, we summarize the recent advances in synthetic work on calothrixins and their analogs.

## 2. Synthesis of Calothrixins

Due to the structural complexity and drug discovery potential, organic and medicinal chemists around the world have embarked on developing efficient synthetic methodologies to produce these natural products. Earlier synthesis of calothrixins was reviewed in 2009 by Satoshi Hibino *et al.* [35]. There has been a lot of progress made in the synthesis of calothrixins and their analogs since then. Herein, the discussion will focus on the progress achieved within the past six years in developing new synthetic routes to achieve the large-scale production of calothrixins.

The calothrixin scaffold consists of five rings (A–E), as shown in Figure 2. Multiple synthetic strategies have been used to synthesize calothrixins starting from suitably substituted indole, quinoline, or carbazole derivatives. The synthetic routes for calothrixins are categorized into three main groups based on the strategy of the last ring closure step in the construction of the calothrixin scaffold (1) ring B closure; (2) ring C closure; and (3) ring D closure (Table 1). No syntheses have been reported in which either ring A or ring E are constructed as the last ring closure step. Recent reports on the synthesis of calothrixins and the key reactions involved in these syntheses are summarized in Table 1.

**Table 1:** Summary of reported syntheses of calothrixin B since 2009.

Research Group	Year	Ring Closure	Key Step	Reference
Velu *et al.*	2014	B	Mn(OAc)_3_ mediated oxidative free radical reaction	[36]
Ishikura *et al.*	2011 & 2012	C	Palladium catalyzed tandem cyclization/cross-coupling	[37,38]
Dethe *et al.*	2014	C	LTA mediated rearrangement of *o*-hydroxy aryl hydrazone	[39]
Nagarajan *et al.*	2014	C	Friedel-Crafts hydroxyalkylation and directed *o*-lithiation	[40]
Mal *et al.*	2014	C	The anionic annulation of MOM-protected furoindolone	[41]
Kusurkar *et al.*	2012	D	Two one pot reaction sequences: a nucleophilic substitution and reduction	[42]
Nagarajan *et al.*	2013	D	Pd catalyzed intramolecular cross-coupling reaction via C–H activation	[43]
Mohanakrishnan *et al.*	2014	D	Electrocyclization of 2-nitroarylvinyl-3-phenylsulfonylvinylindole	[44]
Kumar *et al.*	2014	D	Pd mediated intramolecular C-X/C-H cross coupling reactions	[45]
Hibino *et al.*	2012	D	Allene mediated electrocyclic reaction of the 6π-electron system	[46]
Mohanakrishnan *et al.*	2013	C & D	FeCl_3_ mediated domino reaction of enamines	[47]

### 2.1. Formation of Indole (Rings A and B) as the Last Step in the Construction of the Indolo[3,2-j]Phenanthridine Framework

#### Velu’s Synthesis

**Scheme 1:**
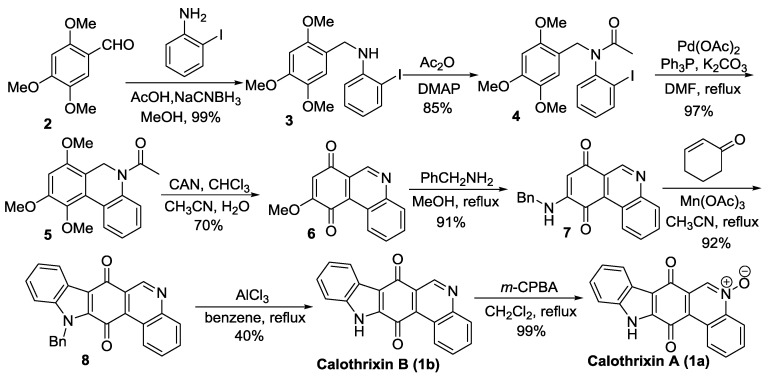
Velu’s synthesis of calothrixins.

In 2014, Velu *et al.* [36] reported a synthesis of calothrixins in which the indole ring is formed last to construct the five-ring scaffold. This approach relies on the construction of the indole ring (rings A and B) on a phenanthridine dione (ring C, D, and E) via a novel oxidative free radical reaction mediated by manganese triacetate Mn(OAc)_3_, as outlined in Scheme 1. The method of Mn(OAc)_3_ mediated oxidative reaction of 2-cyclohexenone with quinones was originally developed by Chuang *et al.* [48,49,50]. None of the existing reports of calothrixin synthesis utilizes the late-stage indole construction strategy. Through this methodology, calothrixin B was synthesized in seven steps, with an overall yield of 19% starting from 2,4,5-trimethoxyindole.

### 2.2. Ring C Closure as the Last Step in the Construction of the Indolo[3,2-j]Phenanthridine Framework

A number of calothrixin syntheses have been reported where the ring C closure was used as the last step in the construction of calothrixin scaffold. This includes the two syntheses reported by Ishikura *et al.* in 2011 and 2012 [37,38], and the three contributions made by Dethe *et al.* [39], Nagarajan *et al.* [40], and Mal *et al.* [41] in 2014.

#### 2.2.1. Ishikura’s Synthesis

**Scheme 2:**
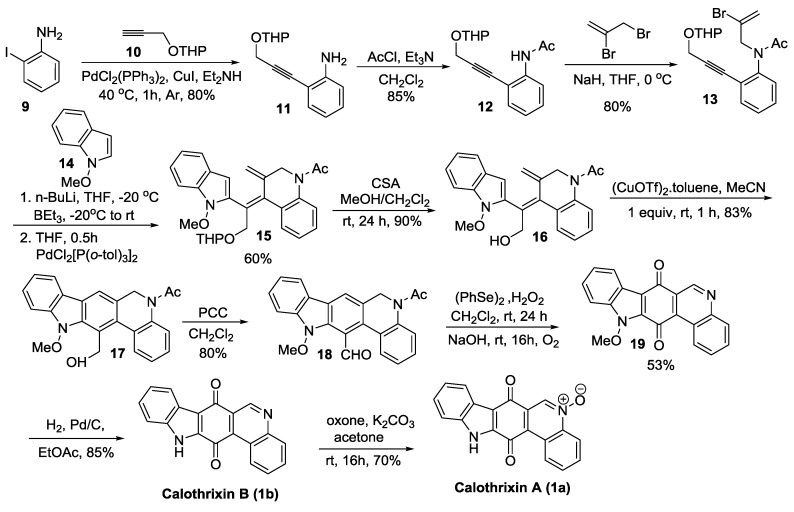
Ishikura’s synthesis of calothrixins.

Ishikura *et al.* [37,38] was one of the first groups that designed a synthetic route to calothrixins in which ring C was cyclized last as shown in Scheme 2. In their ongoing studies of trialkyl(indol-2-yl)borates, they previously found that indolylborates show high reactivity in palladium-catalyzed cross-coupling reactions, such as carbonylative cross-coupling and tandem cyclization/cross-coupling reactions. This new approach for the synthesis of calothrixins A and B was demonstrated through a palladium-catalyzed cross-coupling reaction of 1-methoxyindolylborate (generated *in situ* from 1-methoxyindole by treatment with n-BuLi and BEt_3_) with the intermediate compound **13** to form the compound **15**. Additional novelty of this synthetic route is the strategic use of Cu(OTf)_2_.toluene complex for the 6π-elecrocyclization of the hexatriene intermediate (**16**) to cyclize the ring C to form the compound **17**. Ishikura’s synthesis yielded calothrixin B in nine steps with an overall yield of 9% starting from 2-iodoaniline.

#### 2.2.2. Dethe’s Synthesis

**Scheme 3:**
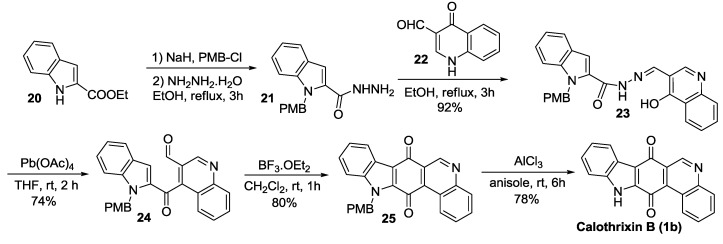
Dethe’s synthesis of calothrixin B.

Dethe *et al.* [39] reported a concise five-step total synthesis of calothrixin B using LTA-mediated rearrangement of a suitable *o*-hydroxy aryl hydrazone into the corresponding quinone as the key step, as outlined in Scheme 3. The synthesis began with the coupling of *N*-PMB (p-methoxy benzyl) protected indolyl hyrazide (**21**) and the quinolinone derivative (**22**) to form the intermediate hydrazone **23**. The hydrazone (**23**) was treated with Pb(OAc)_4_ to undergo the LTA-mediated oxidative rearrangement followed by a BF_3_.OEt_2_-mediated cyclization to form the PMB-protected calothrixin B (**25**). This synthesis is short and high yielding. The overall yield of the calothrixin B synthesis from ethyl indole-2-carboxylate (**20**) is 39% for five steps.

#### 2.2.3. Nagarajan’s Synthesis

**Scheme 4:**
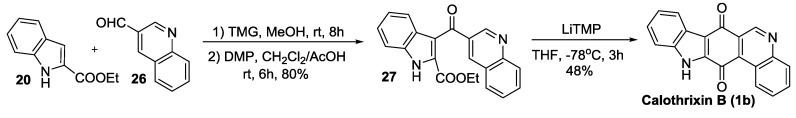
Nagarajan’s synthesis of calothrixin B.

Nagarajan *et al.* [40] outlined a strategy for the synthesis of calothrixin B featuring a directed *o*-metalation reaction as a key step, as outlined in Scheme 4. The synthesis began with the coupling of the commercially available reagents, ethyl indole-2-carboxylate (**20**) and quinoline-3-carboxaldehyde (**26**) in the presence of TMG in MeOH followed by the oxidation of the intermediate product with Dess-Martin periodinane (DMP) in CH_2_Cl_2_/AcOH. This two-step process yielded compound **27**, which was then cyclized by an intramolecular directed *o*-lithiation reaction using lithium tetramethylpiperidide (LiTMP) to form calothrixin B in 48% yield. This synthetic methodology offers a three-step route to calothrixin B from commercially available reagents in an overall yield of 38%.

#### 2.2.4. Mal’s Synthesis

**Scheme 5:**
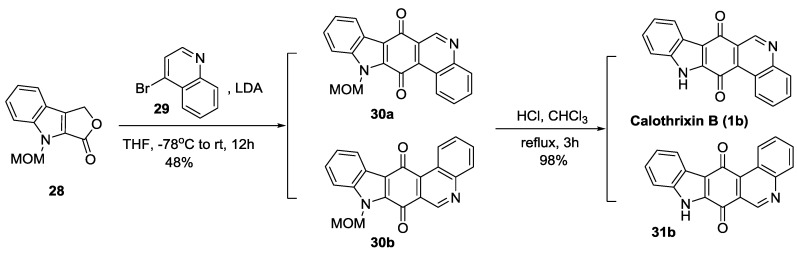
Mal’s synthesis of calothrixin B and its regioisomer.

Mal *et al.* [41] reported a two-step synthesis of calothrixin B from the *N*-protected indololactone (**28**) as outlined in Scheme 5. This reagent was prepared in three simple steps from commercially available α-acetobutyrolactone [51]. Treatment of compound **28** with 4-bromoquinoline (**29**) in the presence of lithium diisopropylamide (LDA) in tetrahydrofuran (THF) at −78 °C afforded an inseparable mixture of compounds **30a** and **30b**. However, after the removal of methoxymethylene (MOM) group using HCl, it gave a mixture of calothrixin B (**1b**) and its regioisomer (**31b**) that could be easily separated in the ratio of 40% and 6%, respectively. This synthetic methodology afforded calothrixin B with an overall yield of 47% for two steps starting from compound **28**.

### 2.3. Ring D Closure as the Last Step in the Construction of the Indolo[3,2-j]Phenanthridine Framework

Another strategy for the calothrixin synthesis involves cyclizing ring D as the last step in the construction of calothrixin scaffold. A number of contributions have been made to this category of syntheses. These include four independent publications by Kusurkar *et al.* in 2012 [42], Nagarajan *et al.* in 2013 [43], Mohanakrishnan *et al.* in 2014 [44], Kumar *et al.* in 2014 [45], and Hibino *et al.* in 2012 [46].

#### 2.3.1. Kusurkar’s Synthesis

**Scheme 6:**
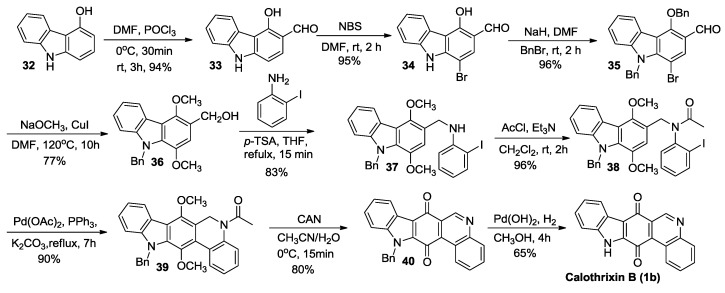
Kusurkar’s total synthesis of calothrixin B.

Kusurkar *et al.* [42] have published a synthesis of calothrixins starting from 4-hydroxy carbazole as outlined in Scheme 6. The novelty of their synthetic route is the unprecedented use of DMF-NaOMe as a reagent for the reduction of the aldehyde group and the use of a high-yielding Pd-catalyzed coupling reaction to construct the ring D of the pentacyclic system. For example, the treatment of compound **35** with NaOMe in dry dimethyl formamide (DMF) and CuI at 120 °C resulted in the substitution of both bromine and benzyloxy groups with methoxy groups with concomitant reduction of the aldehyde resulting in the formation of the compound **36**. Pd-catalyzed cyclization of ring D of the scaffold to form compound **39** is another novel key step in this synthesis. The overall yield for Kusurkar’s synthesis of calothrixin B was 25% for nine steps.

#### 2.3.2. Nagarajan’s Synthesis

**Scheme 7:**
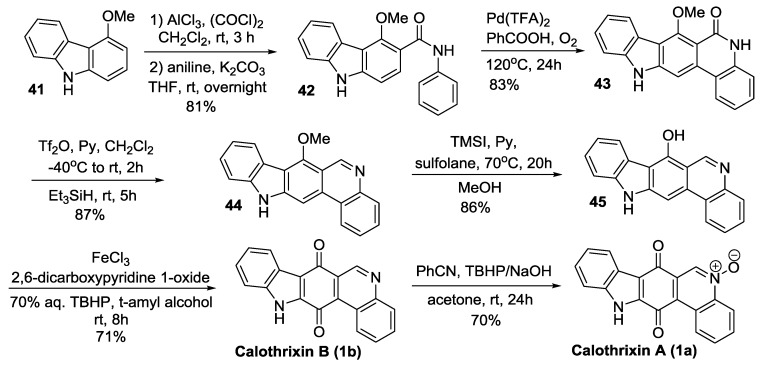
Nagarajan’s synthesis of calothrixins A and B.

In addition to developing a synthetic route for calothrixins that involved the closure of ring C in the last step, Nagarajan *et al.* [43] has also accomplished a synthesis in which ring D was closed last in the preparation of the five-membered ring system, followed by the installation of the necessary substitutions to produce calothrixin B as shown in Scheme 7. This synthesis featured a prominent Pd-catalyzed intramolecular cross-coupling reaction via C-H activation using an appropriately substituted carbazole derivative to construct the indolophenanthridine core ring system of calothrixins. This is the first reported synthesis of calothrixin pentacycles without any protection on the indole N atom. The overall yield for calothrixin B in this Nagarajan’s synthesis is 35% for five steps starting from 4-methoxycarbazole.

#### 2.3.3. Mohanakrishnan’s Synthesis

**Scheme 8:**
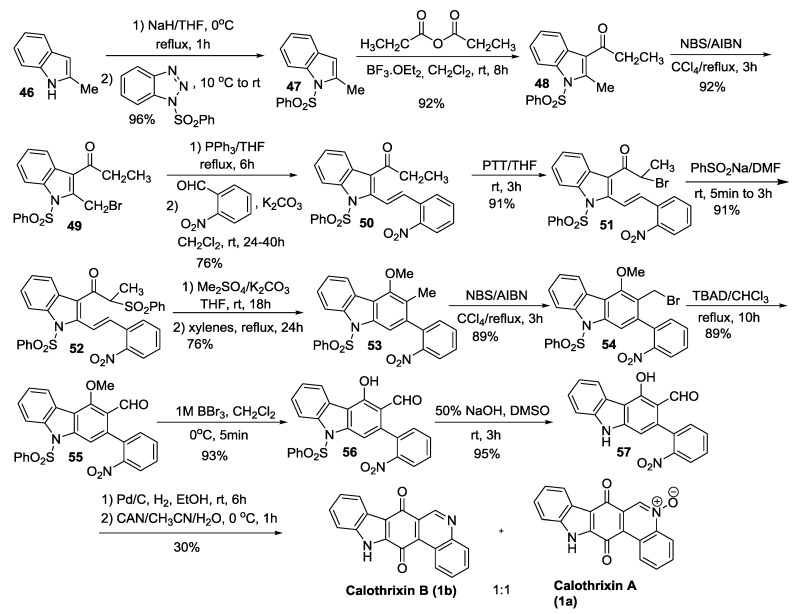
Mohanakrishnan’s synthesis of calothrixins A and B.

Mohanakrishnan *et al.* have reported a linear synthesis of calothrixin B using the thermal electro cyclization of 2-nitroarylvinyl-3-phenylsulfonylvinylindole as the key step as outlined in Scheme 8 [44]. In this key step, the 2,3-divinylidole intermediate generated from compound **52** by treatment with Me_2_SO_4_ was subjected to thermal cyclization in refluxing xylenes to afford the carbazole derivative **53**. Mohanakrishnan’s total synthesis thus afforded calothrixin B from 2-methylindole in 14 steps with an overall yield of about 8%.

#### 2.3.4. Kumar’s Synthesis

**Scheme 9:**
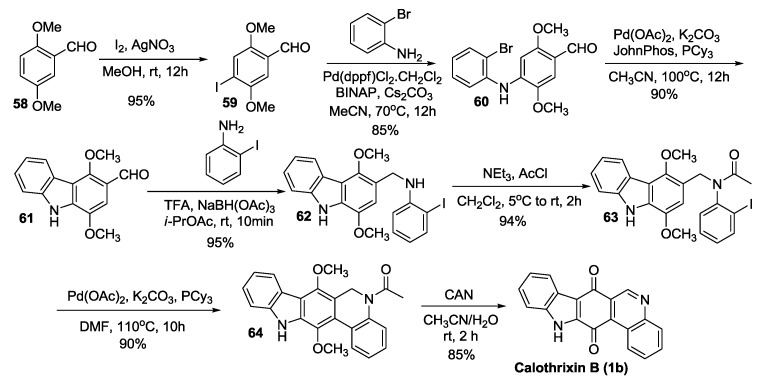
Kumar’s synthesis of calothrixins B.

Kumar *et al.* [45] reported the total synthesis of calothrixin B via multiple palladium-mediated C-X/C-H cross intramolecular coupling reactions as outlined in Scheme 9. The salient feature of this synthesis is the two intramolecular C-X/C-H cross coupling reactions of the intermediates **60** to form compound **61** and **63** to form the compound **64**. The intermediate **60** was cyclized under intramolecular cross coupling reaction condition using Pd(OAc)_2_, PCy_3_, and JohnPhos to afford the carbazole derivative **61**. The cyclization of **63** was carried out using Pd(OAc)_2_, PCy_3_, and K_2_CO_3_ to afford the compound **64.** Kumar’s methodology thus accomplished the synthesis of calothrixin B in 7 steps starting from 2,5-dimethoxybenzaldehyde in an overall yield of 50%.

**Scheme 10:**
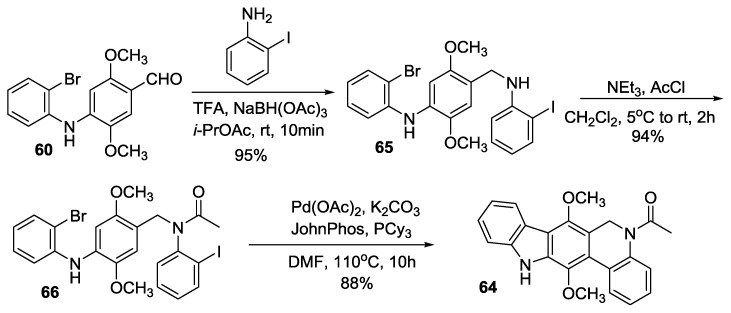
Alternate synthesis of compound **64**.

Compound **60** in Kumar’s synthesis has also been converted to the key intermediate **64** in three steps as shown in Scheme 10. In this synthesis, rings B and D of the calothrixin scaffold were cyclized in one step by intramolecular C-X/C-H cross coupling reaction using Pd(OAc)_2_, PCy_3_, and JohnPhos in the presence of K_2_CO_3_ in DMF to afford the key intermediate **64**.

#### 2.3.5. Hibino’s Synthesis

**Scheme 11:**
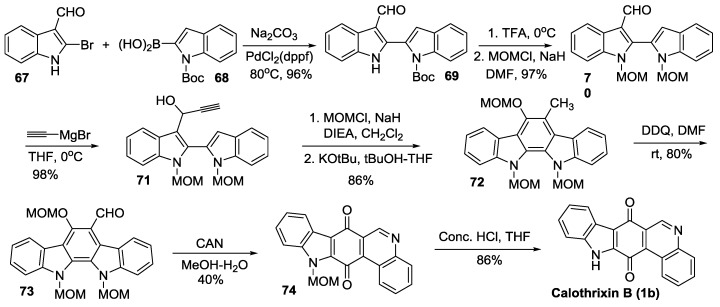
Hibino’s synthesis of calothrixin B.

Hibino *et al.* [46] synthesized calothrixin B and other *N*-alkyl-calothrixins using a biomimetic approach featuring a key allene-mediated electrocyclic reaction of the 6π-electron system, as outlined in Scheme 11. The synthesis of the key intermediate, indolo[2,3-*a*]carbazole (**72**), was carried out by an allene-mediated electrocyclic reaction of MOM-protected compound **71** in the presence of tBuOK in tBuOH-THF. This electrocyclization involved the two [*b*]-bonds of indole units and the allene double bond generated *in situ*. The overall yield for Hibino’s synthesis of calothrixin B from 2-bromoindole-3-carboxaldehyde is 22% for nine steps.

### 2.4. Simultaneous Closure of Rings C and D

Only one methodology of calothrixin synthesis has been developed in which multiple rings were cyclized in a one-pot synthesis. This report was published by Mohanakrisnan *et al.* [47] in 2013, prior to their report in 2014 [44] in which ring D was the last ring closure step.

#### Mohanakrishnan’s Synthesis

**Scheme 12:**
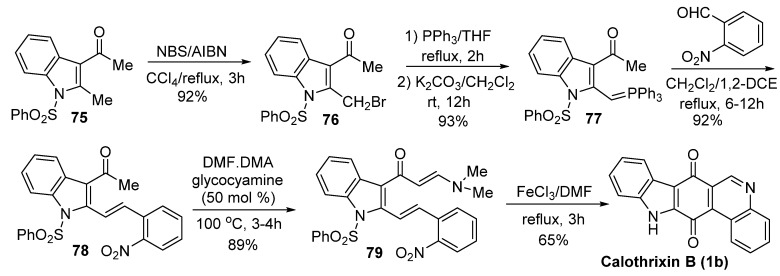
Mohanakrishnan’s synthesis of calothrixins B.

Mohanakrishnan *et al.* [47] reported another novel synthesis of calothrixin B involving a key FeCl_3_ mediated domino reaction of an enamine derivative, as outlined in Scheme 12. In this key step, FeCl_3_ was used to cyclize the key intermediate **79** to afford calothrixin B. Both rings C and D were created in a sigle step in this synthesis. The overall yield of calothrixin B from the compound **75** is 45% for six steps.

## 3. Bioactivities of Calothrixins

The most therapeutically relevant biological activity reported for marine cyanobacterial metabolites is their cytotoxicity. While some antimicrobial studies were also conducted, calothrixins were majorly pursued to serve as novel anticancer molecules. Herein, we summarize all bioactivities conducted on calothrixins and their analogs. Following their isolation, calothrixins quickly gained recognition due to their anticancer activity as preliminary studies revealed a high potency against the human cervical cancer cell line, HeLa cells. Since then, calothrixins have been evaluated against several different cell lines and have been studied for their mode of action. Calothrixins were also evaluated for their antiparasitic activity. A summary of their bioactivities is found below.

### 3.1. Antiparasitic Activity

Along with their discovery in 1999, Smith *et al.* [34] also reported that the cell extracts from cyanobacteria *Calothrix* were found to inhibit the growth of malarial strains. The two calothrixins, calothrixin A and B, were evaluated against the chloroquine (QC)-resistant malarial strain, *P. falciparum* FCR-3 strain (QC-sensitive), and were found to be effective in a dose-dependent manner. The IC_50_ values were observed to be 58 nM and 180 nM, respectively, compared with 83 nM IC_50_ value for chloroquine in the same assay [34].

In an attempt to find a better lead against malaria, Hibino *et al.* [46] synthesized and evaluated several *N*-alkyl calothrixin analogs for their activity against the chloroquine-resistant strain (Table 2). The results for antimalarial activity were compared against chloroquine. Calothrixin B was observed to be the most active (IC_50_ = 120 nM), concurring with the findings of Rickards *et al.* [34]. Calothrixin A was found to be almost equally active (IC_50_ = 185 nM ) as calothrixin B. Although all compounds showed antimalarial activity, substitution of the indole nitrogen atom with various alkyl groups led to a decrease in activity. Among the analogs, 2-hydroxyethyl group showed slightly better activity in comparison to the other alkyl substitutions.

In addition to its antimalarial activities, Smith *et al.* [52,53] reported the antibacterial activity of calothrixin A against *Bacillus subtilis* 168, where it was found to be inhibiting the bacterial growth in a dose-dependent manner without any cell lysis. A complete growth inhibition was achieved at 16 µM concentration.

**Table 2:** Antimalarial activity calothrixin analogs against FCR-3 strain.

Compound	IC_50_ against FCR-3 Strain (nM)	Compound	IC_50_ against FCR-3 Strain (nM)
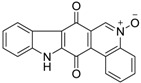	185	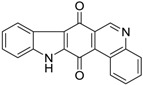	120
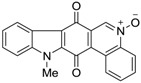	380	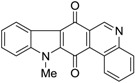	490
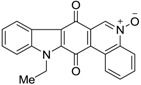	380	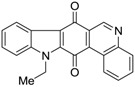	220
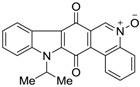	320	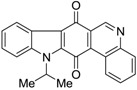	640
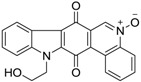	250	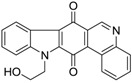	330
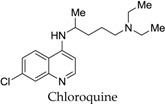	180		

### 3.2. Anticancer Activity

Along with their antimalarial activity, Rickards *et al.* [34] reported calothrixins to be potent against HeLa cells. The corresponding IC_50_ values for calothrixin A and B against the human cervical cancer cell line, HeLa cells, were observed to be 40 nM and 350 nM, respectively. Calothrixins showing similar inhibitory effects against cancer cell lines and malarial strains indicated that they may share a common mode of action. Following this report, in 2003, Waring *et al.* [54] studied the effect of calothrixin A on apoptosis in human Jurkat cancer cells. A well-known inducer of apoptosis, menadione, was used as a positive control to compare the effects of calothrixin A [55]. Calothrixin A was observed to induce cell death via apoptosis in a time- and concentration-dependent manner, and was found to be more potent than menadione in anti-proliferative activity, with IC_50_ values of 1.6 µM and 4.7 µM, respectively (Table 3). IC_50_ values for calothrixin A- and menadione-induced apoptosis were 0.6 µM and 12 µM respectively. Both calothrixin A and menadione were observed to induce cell cycle arrest in the G_2_/M phase, but the concentration required for calothrixin A was much lower than for menadione. The cell cycle arrest in the G_2_/M phase indicated intracellular DNA damage, which was further supported by direct DNA damage observed in cell-free experiments.

Both calothrixin A and menadione were found to be redox active through the observation of additional oxygen intake when added to reductant dithiothreitol (DTT, 2 mM at pH 8.0). Quinones can redox cycle by one or two electron transfers, generating reactive oxygen species (ROS), by which they can damage DNA and induce apoptosis [56]. It was postulated that the ring structure of calothrixin might act as a DNA intercalator that might be responsible for its anticancer activity. In addition, calothrixin A was observed to be cleaving DNA, though less effectively than menadione. Menadione caused significant DNA damage at 10 µM after 60 min incubation, whereas calothrixin A exhibited the same effect at 500 µM.

**Table 3:** Cell cytotoxicities and apoptotic activities of calothrixin A and menadione.

Compound	Structure	IC_50_ for Cytotoxicity	IC_50_ for Apoptosis
Calothrixin A	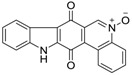	1.6 µM	0.6 µM
Menadione	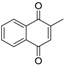	4.7 µM	12 µM

One of the known modes of action for anticancer activities of quinones is by the generation of ROS through redox cycling [56]. In 2004, Wilkes *et al.* [57] carried out a comparative study of bioactivities of calothrixins and some structurally related quinones. This was the first structure–activity relationship study done for calothrixins. The cytotoxic effect of various quinones was evaluated against human cervical cancer cells, HeLa cells, using 3-(4,5-Dimethylthiazol-2-yl)-2,5-diphenyltetrazolium bromide (MTT) assay (Table 4). Calothrixin A and calothrixin B showed cytotoxicity against HeLa cells with EC_50_ values of 0.12 µM and 0.24 µM, respectively. Calothrixin B and ellipticine quinone, which differ by the ring E, showed comparable activities, indicating that there might be only a limited role for the ring E in dictating the bioactivity of calothrixins. Introducing the MOM group on the indole nitrogen seemed to decrease the potency of both calothrixin B and ellipticine quinone with EC_50_ values of 0.42 µM and 0.37 µM, respectively. Further, replacement of the heteroaromatic ring-D with a carbocyclic aromatic ring in ellipticine and its *N*-methoxymethyl analog also led to a decrease in potency, benzocarbazoledione (0.43 µM), and *N*-MOM-benzocarbazoledione (1.6 µM). Menadione showed activity at 3.7 µM. Absence of activity for uncyclized precursors of benzocarbazoledione and *N*-MOM-benzocarbazoledione indicated the importance of a tetracyclic quinone ring structure. In case of all the quinones with EC_50_ < 1.6 µM, the quinones were observed to be reduced to their respective semiquinones, though no direct correlation was observed between the reduction potentials and bioactivity.

**Table 4:** Cytotoxicity of quinones against HeLa cells.

Compound	Structure	EC_50_ (µM) HeLa Cells
Calothrixin A	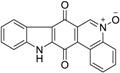	0.12 ± 0.01
Calothrixin B	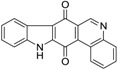	0.24 ± 0.04
*N*-MOM-calothrixin B	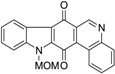	0.42 ± 0.02
Ellipticine quinone	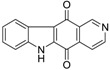	0.15 ± 0.09
*N*-MOM-ellipticine quinone	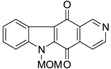	0.37 ± 0.08
Benzocarbazoledione	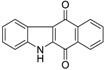	0.43 ± 0.01
*N*-MOM-Benzocarbazoledione	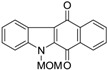	1.6 ± 1.0
Menadione	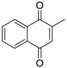	3.7 ± 0.3
Uncyclized precursor to benzocarbazoledione	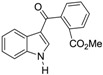	>50
Uncyclized precursor to *N*-MOM-benzocarbazoledione	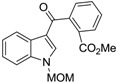	>50

To study the involvement of the ring structure and the mechanism of action in the inhibitory effects, the same group [58] synthesized and analyzed the activity of various simple quinone analogs of calothrixin B (Table 5) in 2007. The compounds were evaluated for their selectivity and antiproliferative activity, using an MTT assay, against three different cell lines: human cervical cancer (HeLa) cells, murine P388 macrophage cancer cells, and simian non-cancerous CV-1 cells.

In this study, calothrixin B was found to be the most potent against HeLa cells (0.25 µM) followed by indolophenanthrene-7,13-dione analog (1.5 µM), indicating the importance of nitrogen in ring D. The activity was further decreased with the deletion of ring E of this analog to form benzoncarbzoledione (1.8 µM). The rest of the analogs showed moderate activity ranging from 7 to 13 µM, while the carbazole-1,4-dione analog was found to be inactive. The results were not as consistent for the P388 cell line, where murrayaquinone (2.3 µM) and 2-methylcarbazoledione (1 µM) were found to be more active compared to calothrixin B (9 µM) or other analogs, whereas calothrixin B and 2-methylcarbazoledione showed similar activity with 2.4 µM and 1.7 µM, respectively, against non-cancerous cell line CV-1. The rest of the analogs had either very low activity or no measurable activity. These studies indicated the importance of rings A–D in the activity of calothrixins. Also, a selective usage of calothrixin B against human cervical cancer cells was indicated by the 10-fold higher effectiveness against HeLa cells (0.25 µM) as compared to CV-1 (2.4 µM) and 38 times more compared to P388 (9 µM). The quinones containing tetra- and penta cyclic systems (calothrixin-B, indolophenanthrene-7,13-dione and benzocarbazole-1,4-dione) were found to be more active against HeLa cells as compared to p388 and CV-1 cells. However, the trend was reversed in the quinones containing bi- and tricylic systems (murrayaquinone, 2-methylcarbazoledione, and isoquinoline-5,8-dione).

In 2009, another group, Hecht *et al.* [59], published a study to determine the mechanism of action and the stage of the cell cycle that is targeted by the calothrixins. Calothrixin A and B and the *N*-methylated derivative were synthesized and tested against CEM leukemia cells to measure their cytotoxicity using an MTT assay. The activity of calothrixin analogs were compared to camptothecin, which is known to cause irreversible DNA damage during the S phase. Delay in S phase due to DNA damage is associated with a block in replication. The IC_50_ value for calothrixin A was found to be five-fold higher than campothecin and the other two analogs were observed to be much less potent, with *N*-methylcalothrixin B being the least at 5 µM compared to calothrixin B at 1 µM (Table 6).

**Table 5:** Antiproliferative activity of calothrixin B and analogs in MTT assay against HeLa cells, P388 cells, and CV-1 cells.

Compound	Structure	EC_50_ (µM) HeLa Cells	EC_50_ (µM) P388 Cells	EC_50_ (µM) CV-1 Cells
Calothrixin B	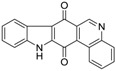	0.25 ± 0.05	9 ± 2	2.4 ± 0.7
Indolophenanthrene-7,13-dione	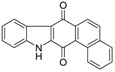	1.5 ± 0.3	>50	>50
Benzocarbzoledione	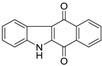	1.8 ± 0.1	>50	>50
Carbazole-1,4-dione	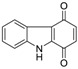	>50	>50	>50
Murrayaquinone	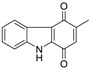	13 ± 1	2.3 ± 0.3	10 ± 2
2-Methylcarbazoledione	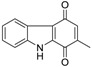	7 ± 1	1.0 ± 0.1	1.7 ± 0.4
Isoquinoline-5,8-dione	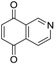	12 ± 1	9 ± 2	>50

The effect on the cell cycle was studied for these compounds using a mitotic inhibitor nocodazole, which blocks re-entry of cells into the G_1_ phase. Calothrixin B was observed to arrest the G_1_ phase at 0.1 μM concentrations, whereas calothrixin A and *N*-methylcalothrixn B showed no effects at the same concentration. At higher concentrations, calothrixin A and *N*-methylcalothrixn B led to cell accumulation in S and G_2_/M phase. Compared to camptothecin, these effects were found to be readily reversible. Calothrixins were also evaluated for their activity against topoisomerase I. The effects of calothrixins were studied keeping camptothecin as a positive control, which is a known topoisomerase I poison [60]. It was observed that calothrixins A, B, and *N*-methylcalothrixin B were capable of stabilizing the covalent topoisomerase I/DNA complex at 18%, 13%, and 11%, respectively, compared to 100% at 5 µM concentration of camptothecin.

Calothrixins and their analogs were observed to be affecting different stages of cell cycle in a reversible manner, leading to low micromolar range cytotoxicities. Thus, calothrixins have shown a wide array of activity including antimalarial, anticancer, and antibacterial. The modes of action of calothrixins’ bioactivities have not been fully deciphered yet.

**Table 6:** Cytotoxicity effects against CEM leukemia cell line and topoisomerase I inhibition.

Compound	Structure	IC_50_ (µM) in CEM Leukemia Cells	% Topo I DNA Cleavage at 5 µM	% NaCl Induced Reversibility at 5 µM
Camptothecin	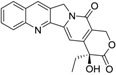	0.04 ± 0.01	100	100
Calothrixin A	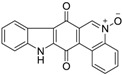	0.20 ± 0.02	18	17
Calothrixin B	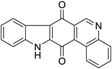	1.05 ± 0.30	13	16
*N*-methycalothrixin B	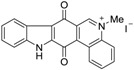	5.13 ± 0.72	11	13

## 4. Conclusions

While the medicinal and biosynthetic potential of terrestrial plants and microbes is fairly well studied, comparatively little is known regarding the chemistry and biological activity of organisms in the marine environment. A unique group of oxygenic photosynthetic bacteria known as cyanobacteria populate diverse habitats throughout the world. Their potential as a good source of new therapeutic lead compounds has been realized during the last few decades. Calothrixins, which are cyanobacterial metabolites, have demonstrated a diverse range of bioactivities that include antimalarial, anticancer, and antibacterial properties. They have been observed to target various aspects of RNA synthesis in bacteria. Further investigation of the exact mechanism for their bioactivity is still required for many analogs, which will be beneficial for the ongoing development and lead optimization. Several research groups have developed synthetic routes to obtain these natural products. This review emphasized the synthetic progress accomplished within the past six years in developing new synthetic routes. Since 2009, 11 novel syntheses have been published to improve upon the efficiency in the production of calothrixins. The number of steps in the various synthetic strategies ranges from 2 to 14, while the overall yield to construct calothrixin B ranges from 8% to 50%. Thus, significant progress has been made in the last decade in attaining more efficient synthesis of calothrixins, which may aid to establish calothrixins as a potential anti-cancer candidate in the future.

## Abbreviations

AcCl: Acetyl chloride
Ac_2_O: Acetic anhydride
AcOEt: Ethyl acetate
AcOH: Acetic acid
AIBN: Azobisisobutyronitrile
AlCl_3_: Aluminum chloride
BEt_3_: Triethylborane or triethylboron
BF_3_.OEt_2_: Boron trifluoride diethyl etherate
CAN: Ceric ammonium nitrate
CCl_4_: Carbon tetrachloride
CH_2_Cl_2_: Dichloromethane
CH_3_CN: Acetonitrile
COSY: Correlation spectroscopy
c-PC: c-Phycocyanin
CSA: Camphorsulfonic acid
CuI: Copper(I) iodide
(CuOTf)_2_.toluene: Trifluoromethanesulfonate toluene complex
DMAP: 4-Dimethylaminopyridine
DMF: Dimethylformamide
DMP: Dess-Martin periodinane
DMSO: Dimethyl sulfoxide
DoM: Directed *o*-metalation
DTT: Dithiothreitol
Et_2_NH: Diethylamine
Et_3_SiH: Triethylsilane
FeCl_3_: Iron(III) chloride
HCO_2_H: Formic acid
HDACs: Histone deacetylases
HIV: Human immunodeficiency virus
H_2_O_2_: Hydrogen peroxide
K_2_CO_3_: Potassium carbonate
LDA: Lithium diisopropylamide
LiTMP: Lithium tetramethylpiperidide
LTA: Lead tetracetate
MeOH: Methanol
Mn(OAc)_3_: Manganese triacetate
MOM: Methoxymethyl protecting group
MTT: 3-(4,5-Dimethylthiazol-2-yl)-2,5-diphenyltetrazolium bromide
NaCNBH_3_: Sodium cyanoborohydride
NaH: Sodium hydride
NaOH: Sodium hydroxide
NaOMe: Sodium methoxide
NBS: *N*-bromosuccinimide
*n*-BuLi: *n*-Butyllithium
NH_2_NH_2_.H_2_O: Hydrazine hydrate solution
Pb(OAc)_4_: Lead(IV) acetate
PCC: Pyridinium chlorochromate
Pd/C: Palladium on carbon
PdCl_2_(PPh_3_)_2_: Bis(triphenylphosphine)palladium(II) dichloride
Pd_2_(dba)_3_: Tris(dibenzylideneacetone)dipalladium(0)
Pd(dppf)Cl_2_: [1,1′-Bis(diphenylphosphino)ferrocene] dichloropalladium(II)
Pd(OAc)_2_: Palladium acetate
Pd(TFA)_2_: palladium(II) trifluoroacetate
PhCN: Benzonitrile
(PhSe)_2_: Diphenyl diselenide
PMB: *p*-Methoxybenzyl
PMBCl: *p*-Methoxybenzyl chloride
P(*o*-tol)_3_: Tri(*o*-tolyl)phosphine
POCl_3_: Phosphoryl chloride
PPh_3_: Triphenylphosphine
*p*-TSA: *p*-Toluenesulfonic acid
PTT: Polytrimethylene terephthalate
rac-BINAP: Racemic 2,2′-bis(diphenylphosphino)-1,1′-binaphthalene
ROS: Reactive oxygen species
TBAD: Bis(tetrabutylammonium) dichromate
TBHP: *Tert*-butyl hydroperoxide
Tf_2_O: Trifluoromethanesulfonic anhydride
THF: Tetrahydrofuran
THP: Tetrahydropyran
TMG: 1,1,3,3-Tetramethylguanidine
TMP: 2,2,6,6-Tetramethylpiperidine
TMSI: Trimethylsilyl iodide
